# Prevalence and Clinical Impact of Incidental Findings on Preoperative 3D Planning Computed Tomography for Total Shoulder Arthroplasty

**DOI:** 10.5435/JAAOSGlobal-D-21-00291

**Published:** 2022-08-05

**Authors:** Yuqing Chen, Sarav S. Shah, Alexander M. Roche, Lambert T. Li, Matthew Chilton, Benjamin Saks, Meghan Macaskill, Glen Ross

**Affiliations:** From the New England Baptist Hospital, Boston, MA.

## Abstract

**Introduction:**

3D planning software for shoulder arthroplasty recently emerged for aiding in intraoperative determination of native glenoid. These protocols often require increased scan resolution, however, raising the question of an increased prevalence and clinical impact of incidental findings (IFs) from preoperative imaging.

**Methods:**

A retrospective review of preoperative shoulder CT reports was conducted for 333 consecutive patients planning anatomic or reverse total shoulder arthroplasties. Patients with thin-sliced CT scans (1.25 mm) were compared with those with standard CT scans (2.5 mm). Poisson regression was performed with baseline characteristics and potentially pathologic IFs (PPIFs).

**Results:**

IFs were present in 131 of the 333 scans (39.3%), and 38 of the 333 scans (11.4%) included PPIFs. Only 8 of the 333 scans (2.4%) required workup, with 2 of the 333 (0.6%) leading to new cancer diagnoses. Thin-sliced CT scans detected a higher mean number of IFs (1.12 versus 0.22, *P* < 0.001) while the mean number of PPIFs remained similar (0.13 versus 0.10, *P* = 0.43).

**Conclusion:**

IFs are frequent; however, only 0.6% scans led to new cancer diagnoses. Comparison of thin-sliced with standard CT scans revealed a higher frequency of IFs but similar PPIFs, indicating increased burden of IFs without the benefit of identifying additional malignancies. As demand rises for shoulder arthroplasties, surgeons should consider the potential hidden costs of IFs when using 3D planning programs.

CT of the shoulder is important in the preoperative assessment of glenoid morphology in anatomic total shoulder arthroplasties (aTSAs) and reverse shoulder arthroplasties (rTSAs).^1,2^ The volume of preoperative CT scans is likely to rise as demand for aTSAs and rTSAs increases,^3-5^ especially with the development of 3D planning software, which is of current industry interest and evaluation.^6^ 3D planning software has recently emerged, specifically to help with difficulty in intraoperative determination of native glenoid positioning secondary to variability of bony morphology.^7^ Furthermore, 3D imaging and templating for shoulder arthroplasty provides information to the surgeon that allows for more accuracy and less variable decision making at the time of surgery.^8,9^ 3D planning software also plays a critical role in clinical decision making such as implant selection^6^ and achieving a desired implant position with^10^ or without patient-specific instrumentation.^11^ A typical CT scan protocol for 3D planning calls for a thinner slice thickness of 1.25 mm, which is more refined compared with typical scans which are 2.5 mm thick.^12^ The unintended consequence of higher volumes and increased resolution of imaging associated with all the various 3D planning software's protocols is the clinical and financial effect of their associated incidental findings (IFs).

IFs have grown with increased utilization of high-resolution imaging over the years, presenting mounting challenges for providers and patients over the question of management and counseling.^13^ These implications are broad, ranging from changes in disease incidence to increased utilization of resources.^13,14^ A survey of orthopaedic surgeons found that 96% of respondents reported practicing defensive medicine.^15^ Although IFs can lead to diagnosis and treatment for certain patients, the physical and emotional harms of the clinical workup cascade that follows must also be considered.^16,17^

IFs have already been characterized across various organ systems and imaging modalities.^18-20^ Of note, a recent study found that the highest percentage of IFs were found on chest CT, which holds relevance for imaging of the shoulder, and there exists a paucity of studies on IFs in joint imaging.^21^ A recent study reported IFs in 45.7% of preoperative images for total hip and knee arthroplasties,^22^ leaving incidentalomas in preoperative shoulder imaging a gap in knowledge to be pursued.

The purpose of this study was to elucidate the prevalence and clinical impact of IFs noted on preoperative CT imaging used in 3D planning using thinner slices versus standard CT imaging thickness slices for aTSAs and rTSAs. Specifically, we sought to identify the most common IFs and investigate the clinical course associated with each finding and the need for additional testing or delay in surgery.

## Methods

### Incidental Finding Categorization

To minimize variation in classification, the definition of “incidental finding” and various subgroup terminologies follows previously published literature.^23,24^ An IF is defined as any observation in the final clinical imaging report that does not pertain to the glenohumeral joint or acromioclavicular joint. Similar to previous studies, nonpathological anatomic variations, postsurgical changes, and contralateral shoulder observations on scout images are excluded.^22,23^

IFs were further categorized as either asymptomatic or potentially pathologic. Asymptomatic IFs are defined as IFs which are considered benign and do not mention need for follow-up in the radiology report.^25,26^ Potentially pathologic IFs (PPIFs) were defined as any finding that could represent malignancy or otherwise require additional clinical workup. All reports in which the radiologist discusses pathologic consideration and a need for follow-up were considered PPIFs. The actual clinical impact of all PPIFs was assessed by a review of medical records for clinical workup prompted by PPIFs, including imaging studies, consultations, laboratory testing, biopsies, surgical procedures, or other diagnostic procedures.

### Data Source #1: Institutional Data

IRB approval was granted by New England Baptist Hospital before the beginning of this study. This retrospective chart review study comprised 333 consecutive patients identified from a single surgeon's radiology database (G.R.). Patients undergoing a preoperative shoulder CT scan between January 2013 and December 2019 were included, regardless of whether they ultimately received aTSA or rTSA. Revision procedures were excluded to avoid duplication of data. Preoperative shoulder CT reports were reviewed for IFs and classified according to the abovementioned definitions. Both institutional records and external primary care physician records were obtained for follow-up analysis on PPIFs, dating from the time of preoperative imaging through June 2020 to ensure at least six months of follow-up time. These records comprised office visits, surgical notes, laboratory reports, and imaging reports. Records were deemed inaccessible if at least four attempts were made to contact multiple primary care physician offices, third party record departments, and the patient for their records, until all points of contact were exhausted.

### Statistical Methods

Demographic variables at baseline including patient age; sex; type of planned shoulder arthroplasty; smoking status; BMI more than 30; and medical history including diabetes mellitus, hypertension, cardiovascular disease, cancer, renal disease, COPD, and liver disease are summarized in Table 1. Patients were further divided into two groups according to CT slice thickness: standard (2.5 mm) or thin (1.25 mm). Descriptive statistics for these groups are presented in Table 2. The presence and total number of IFs and PPIFs were tabulated and categorized in Table 3.

**Table 1:** Baseline Demographics Overall

Demographic Variable	N (%)
Patients	333
Operation (aTSA/rTSA)	292/41
Age (y), mean ± SD	66.2 ± 8.9
Male sex	191 (57)
BMI > 30	116 (35)
Current smoker	15 (5)
Diabetes mellitus	41 (12)
Hypertension	125 (38)
History of cardiovascular disease	64 (19)
History of cancer	64 (19)

aTSA = anatomic total shoulder arthroplasty; rSTA = reverse shoulder arthroplasty

**Table 2:** Baseline Demographics in Thin and Standard CT scans

Parameter	N	CT slice thickness		*P*
		Standard (2.5 mm)	Thin (1.25 mm)	
Sex				
Male	191	58.0%	56.8%	0.827
Female	142	42.0%	43.2%	
Diabetes	41	11.9%	12.6%	0.838
Hypertension	124	38.5%	36.3%	0.688
Current smoker	15	1.4%	6.8%	**0.018**
BMI >30	116	32.2%	36.8%	0.376
History of cancer	64	24.5%	15.3%	**0.035**
History of cardiovascular disease	68	14.0%	25.3%	**0.012**
Age (mean, 95% CI)		64.7 (63.3-66.2)	67.2 (66.0-68.5)	**0.011**

**Table 3:** IFs and PPIFs by Category

IF Category	Number of IFs (%)	Number of PPIFs (%)
Pulmonary	131 (53.9%)	31 (81.6%)
Skeletal	53 (20.0%)	1 (0.4%)
Cardiovascular	40 (16.5%)	0 (0%)
Thyroid	10 (4.1%)	2 (5.3%)
Lymph	8 (3.3%)	3 (7.9%)
Breast	1 (0.4%)	1 (0.4%)
Total	243 (100%)	38 (100%)

IF = incidental finding; PPIF = potentially pathologic IF

Regression analysis was conducted using a Poisson model, initially including all demographic variables as predictor variables and PPIF count as the outcome variable. In subsequent models, nonstatistically significant predictor variables were removed in order of the highest *P*-value until only significant variables were left in the final model. Age and sex variables were included for the purpose of interest and comparison. All analyses were conducted in SPSS V26.0, and statistical significance was set at 0.05.

## Results

### Baseline Characteristics

A total of 333 preoperative shoulder CT scans were included in this study. Most of the CT scans were obtained in preparation for aTSA in 292 of the 333 cases (87.7%), with the remaining 41 of the 333 cases (12.3%) for rTSA. Most patients were male at 191 of the 333 cases (57.4%), and 142 of the 333 cases (42.6%) were female. The mean age of patients was 66.2 years (range 42 to 88). There were 143 CT scans of 2.5 mm thickness and 190 scans of 1.25 mm thickness. Baseline characteristics comparison is summarized in Table 2.

### Frequency of Incidental Findings

Of the 333 shoulder CT scans, 131 (39.3%) reported at least one IF. Of those, 38 reports contained PPIFs, comprising 38 of all the 333 shoulder CT scans (11.4%) . To account for reports with multiple IFs, the frequency of IFs was totaled at 243, with 206 of the 243 (84.8%) classified as asymptomatic IFs and 38 of the 243 (15.6%) classified as PPIFs. The mean number of IFs in thin-sliced CT scans was significantly higher than that in thick-sliced CT scans, at 1.12 versus 0.22, *P* < 0.001. The mean number of PPIFs, however, was not significantly different between the two groups (0.13 versus 0.10, *P* = 0.434, Table 4) for thin and thick-sliced CT scans, respectively.

**Table 4:** Number of Incidental Findings on Thin Versus Standard Thickness CT scans

Mean number of IFs					
	N	Mean	95% Confidence Interval for Mean		*P*
			Lower Bound	Upper Bound	
Standard (2.5 mm)	143	0.22	0.13	0.30	**<0.001**
Thin (1.25 mm)	190	1.12	0.93	1.30	

Mean number of PPIFs					
	N	Mean	95% Confidence Interval for Mean		*P*-value
			Lower Bound	Upper Bound	
Standard (2.5 mm)	143	0.10	0.05	0.15	0.434
Thin (1.25 mm)	190	0.13	0.08	0.18	

PPIF = potentially pathologic IF

Most IFs were pulmonary findings in 131 of the 243 cases (53.9%) and degenerative skeletal changes findings that did not pertain to the glenohumeral joint or acromioclavicular joint in 53 of the 243 IFs (20.0%). The remaining findings were cardiovascular in 40 of the 243 cases (16.5%), thyroid in 10 of the 243 cases (4.1%), lymph in 8 of the 243 cases (3.3%), and breast in 1 of the 243 cases (0.4%). The most common types of PPIFs were pulmonary nodules, which accounted for 20 of the 38 PPIFs (52.6%). Other pulmonary pathologies included ill-defined opacities of concern for infectious or inflammatory pathologies contributing to 11 of the 38 PPIFs (28.9%). The remaining PPIFs comprised three enlarged lymph nodes (7.9%), two thyroid nodules (5.2%), one breast tissue density (2.6%), and one intramedullary density (2.6%).

### Actual Clinical Impact

Of the 38 PPIF cases, four were excluded secondary to inaccessible primary care physician records. Of the 34 PPIFs with accessible primary care records, only eight PPIFs (8/34, 23.5%) documented follow-up prompted exclusively by the preoperative shoulder CT. Of the eight PPIFs, four were found in the thin CT group and four in the thick CT group. The remaining PPIFs were already under observation, under routine lung cancer screening, or otherwise not noted to have any effect on the clinical course. The clinically impactful PPIFs included four pulmonary nodules, two lung consolidations, one enlarged lymph node, and one humeral intramedullary lesion. All eight cases required additional imaging, including a total of two chest radiographs, eight chest CT scans, one neck CT scan, and one shoulder MRI scan. Because of follow-up, two cases led to new cancer diagnoses; one case was investigated for possible chronic infectious bronchiolitis; and the remainder of the PPIFs were ruled benign with no additional workup. The two cases of cancer both received additional imaging, consultations, biopsy, and surgery, leading to diagnosis of lung adenocarcinoma and additional treatment. One of these cases experienced a delay in shoulder arthroplasty, but after receiving clearance from the pulmonologist, the patient successfully completed their TSA. No deaths were recorded over the course of follow-up in this study.

### Regression Analysis

In the analysis of PPIFs, current smoking status (IRR = 3.95, *P* = 0.005) was significantly associated with higher risk for increasing PPIFs. This corresponds with the fact that 31 of the 38 PPIFs (81.6%) were pulmonary, and smoking has long been an established risk factor for pulmonary conditions such as lung cancer.^25^ The association of smoking with higher likelihood of PPIFs has also been identified in a study by Orme et al.^24^

## Discussion

Our results indicate that IFs are frequent, with 39.3% of the shoulder CT scans (131/333) reporting IFs; however, potentially pathologic findings were only found in 11.4% of the reports (38/333), and 0.6% (2/333) led to a new cancer diagnosis. The typical CT scan protocol for 3D planning calls for thinner slice thickness (1.25 mm), which is more refined compared with typical scans which are 2.5 mm thick.^12^ Thin-sliced CT scans detected higher frequencies of IFs than standard CT scans, but the frequency of PPIFs did not differ between the groups. Furthermore, there is an indirect clinical impact on the patient. The daunting responsibilities of a clinical workup that follow indeterminate findings can take a physical and emotional drain on the patient. Overall, our results demonstrate the clinical burden of IF management seen with the increased resolution imaging associated with all 3D planning software's protocols without an added benefit of detecting additional malignancy.

The results of our study combined with the growing trajectory of shoulder arthroplasty volumes and increased use of 3D planning software suggest that IFs, and thus associated clinical burden, are likely to increase. Olaiya et al^27^ noted that there is a lack of studies on clinical parameters of 3D planning, such as time or cost, and another study demonstrated wide variability in total shoulder arthroplasty planning software measurements compared with a control CT-derived 3D-printed scapula.^28^ Although there is currently not enough evidence to support the use of 3D planning software over 2D CT planning, there is potential in its utility for complex cases and revisions, as well as for surgeons in training or with limited experience.^6,27^ Our study sheds light on the potential hidden costs and clinical impact of IFs, especially using 3D planning CT protocols.

It is important to consider that the management of IFs encompasses a wide variety of factors and is not limited to purely procedural workup costs. Radiology report recommendations phrased as “correlate clinically” or “if patient is high risk, follow up,”^29^ require additional investigation and time to reach a decision based on patient history and the provider's and patient's unique tolerance of risk. Even when a report does not yield substantial findings, repeat imaging may be recommended to assess for stability, subjecting the patient to additional uncertainty and turmoil. Hence, emotional burden must also be considered. From the patient's perspective, the responsibilities of a clinical workup that follow indeterminate findings can take a physical and emotional drain because diagnostic procedures can comprise radiation, invasive procedures, or other harmful exposures that may offset the benefit.^16,17^

Preoperative imaging can provide valuable information to the surgeon for planning arthroplasty, and it is understandably frustrating for patients and providers to manage unexpected, usually benign IFs. Although identification of malignancies was infrequent in this study, it is important to avoid hindsight bias and acknowledge the reality of uncertainty under most circumstances. In considering the results of this study, surgeons may find it useful to pay particular attention to patients with pulmonary findings because these were the most common types of IFs and the most concerning for potential malignancy and warrant additional follow-up. Surgeons should also be aware that increased scan resolution in 3D planning can hold unintended consequences in detecting additional IFs because resolution is especially substantial for small-volume structures such as lung nodules.^31^ At the same time, a notable limitation of our study was that there were baseline characteristics differences between the two groups of comparison, which may be a confounding variable. There was no difference in PPIFs between the groups, however, thus mitigating concern for confounding.

There were other limitations to this study. First, the exclusion of unattainable primary care physician records decreased the sample size for PPIF and clinical impact investigation. However, only four PPIFs (4/38, 10.5%) were excluded after at least four attempts at attaining information and through all possible avenues of communication before ceasing. Another limitation is that our study did not analyze CT scans thinner than 1.25 mm, whereas the thinnest slices in 3D planning protocols are around 0.625 mm.^12^ Finally, we did not quantify the emotional or psychological burden of IFs, which may add to the social burden and could be an area of future research.

## Conclusion

This study investigates the prevalence, clinical impact, and cost of IFs on preoperative shoulder CT. Although IFs were frequent in the evaluation of preoperative shoulder CT scans, only 0.6% (2/333 scans) led to a new cancer diagnosis. Comparison of thin-sliced CT scans associated with 3D planning software with standard CT scans revealed a higher frequency of IFs but similar frequency of PPIFs, indicating that there is increased burden of IFs without the benefit of identifying additional malignancies. In the context of rising demand for shoulder arthroplasties and 3D planning programs, surgeons should be aware of the potential hidden costs and clinical impact of IFs.

## References

[1] Friedman RJ Hawthorne KB Genez BM: The use of computerized tomography in the measurement of glenoid version. J Bone Joint Surg Am 1992;74:1032-1037. 1522089

[2] Gates S Sager B Khazzam M: Preoperative glenoid considerations for shoulder arthroplasty: A review. EFORT Open Rev 2020;5:126-137. 32296546 10.1302/2058-5241.5.190011 PMC7144890

[3] Bixby EC Boddapati V Anderson MJJ Mueller JD Jobin CM Levine WN: Trends in total shoulder arthroplasty from 2005 to 2018: Lower complications rates and shorter lengths of stay despite patients with more comorbidities. JSES Int 2020;4:657-661. 32939502 10.1016/j.jseint.2020.04.024 PMC7479025

[4] Day JS Lau E Ong KL Williams GR Ramsey ML Kurtz SM: Prevalence and projections of total shoulder and elbow arthroplasty in the United States to 2015. J Shoulder Elbow Surg 2010;19:1115-1120. 20554454 10.1016/j.jse.2010.02.009

[5] Palsis JA Simpson KN Matthews JH Traven S Eichinger JK Friedman RJ: Current trends in the use of shoulder arthroplasty in the United States. Orthopedics 2018;41:e416-e23. 29658976 10.3928/01477447-20180409-05

[6] Werner BS Hudek R Burkhart KJ Gohlke F: The influence of three-dimensional planning on decision-making in total shoulder arthroplasty. J Shoulder Elbow Surg 2017;26:1477-1483. 28162884 10.1016/j.jse.2017.01.006

[7] Iannotti JP Greeson C Downing D Sabesan V Bryan JA: Effect of glenoid deformity on glenoid component placement in primary shoulder arthroplasty. J Shoulder Elbow Surg 2012;21:48-55. 21600787 10.1016/j.jse.2011.02.011

[8] Youderian AR Iannotti JP: Preoperative planning using advanced 3-dimensional virtual imaging software for glenoid component in anatomic total shoulder replacement. Tech Shoulder Elbow Surg 2012;13:145-150.

[9] Scalise JJ Codsi MJ Bryan J Brems JJ Iannotti JP: The influence of three-dimensional computed tomography images of the shoulder in preoperative planning for total shoulder arthroplasty. J Bone Joint Surg Am 2008;90:2438-2445. 18978413 10.2106/JBJS.G.01341

[10] Jacquot A Gauci MO Chaoui J : Proper benefit of a three dimensional pre-operative planning software for glenoid component positioning in total shoulder arthroplasty. Int Orthop 2018. 10.1007/s00264-018-4037-1 29968136

[11] Iannotti JP Weiner S Rodriguez E : Three-dimensional imaging and templating improve glenoid implant positioning. J Bone Joint Surg Am 2015;97:651-658. 25878309 10.2106/JBJS.N.00493

[12] Tornier, Blueprint 3D Planning + PSI. Wright-Medical. http://www.wrightemedia.com/ProductFiles/Files/PDFs/AP-013380_EN_LR_LE.pdf. Accessed April 6, 2021.

[13] Ding A Eisenberg JD Pandharipande PV: The economic burden of incidentally detected findings. Radiol Clin North Am 2011;49:257-265. 21333777 10.1016/j.rcl.2010.11.004 PMC3094927

[14] Berlin L: The incidentaloma: A medicolegal dilemma. Radiol Clin North Am 2011;49:245-255. 21333776 10.1016/j.rcl.2010.11.002

[15] Sethi MK Obremskey WT Natividad H Mir HR Jahangir AA: Incidence and costs of defensive medicine among orthopedic surgeons in the United States: A national survey study. Am J Orthop 2012;41:69-73. 22482090

[16] Brown SD: Professional norms regarding how radiologists handle incidental findings. J Am Coll Radiol 2013;10:253-257. 23545084 10.1016/j.jacr.2012.10.003

[17] Casarella WJ: A patient's viewpoint on a current controversy. Radiology 2002;224:927. 12202734 10.1148/radiol.2243020024

[18] Flor N Di Leo G Squarza SA : Malignant incidental extracardiac findings on cardiac CT: Systematic review and meta-analysis. AJR Am J Roentgenol 2013;201:555-564. 23971446 10.2214/AJR.12.10306

[19] Frank L Quint LE: Chest CT incidentalomas: Thyroid lesions, enlarged mediastinal lymph nodes, and lung nodules. Cancer Imaging 2012;12:41-48. 22391408 10.1102/1470-7330.2012.0006 PMC3335330

[20] Jacobs PC Mali WP Grobbee DE van der Graaf Y: Prevalence of incidental findings in computed tomographic screening of the chest: A systematic review. J Comput Assist Tomogr 2008;32:214-221. 18379305 10.1097/RCT.0b013e3181585ff2

[21] O'Sullivan JW Muntinga T Grigg S Ioannidis JPA: Prevalence and outcomes of incidental imaging findings: Umbrella review. BMJ 2018;361:k2387. 29914908 10.1136/bmj.k2387 PMC6283350

[22] Hassebrock JD Makovicka JL Clarke HD Spangehl MJ Beauchamp CP Schwartz AJ: Frequency, cost, and clinical significance of incidental findings on preoperative planning images for computer-assisted total joint arthroplasty. J Arthroplasty 2020;35:945-949 e1. 31882348 10.1016/j.arth.2019.11.030

[23] Orme NM Fletcher JG Siddiki HA : Incidental findings in imaging research: Evaluating incidence, benefit, and burden. Arch Intern Med 2010;170:1525-1532. 20876402 10.1001/archinternmed.2010.317 PMC3721142

[24] Orme NM Wright TC Harmon GE : Imaging Pandora's box: Incidental findings in elderly patients evaluated for transcatheter aortic valve replacement. Mayo Clin Proc 2014;89:747-753. 24943693 10.1016/j.mayocp.2014.03.011

[25] MacMahon H Naidich DP Goo JM : Guidelines for management of incidental pulmonary nodules detected on CT images: From the Fleischner Society 2017. Radiology 2017;284:228-243. 28240562 10.1148/radiol.2017161659

[26] Munden RF Carter BW Chiles C : Managing incidental findings on thoracic CT: Mediastinal and cardiovascular findings. A white paper of the ACR incidental findings committee. J Am Coll Radiol 2018;15:1087-1096. 29941240 10.1016/j.jacr.2018.04.029

[27] Olaiya OR Nadeem I Horner NS : Templating in shoulder arthroplasty—a comparison of 2D CT to 3D CT planning software: A systematic review. Shoulder Elbow 2020;12:303-314. 33123220 10.1177/1758573219888780 PMC7545523

[28] Shah SS Sahota S Denard PJ : Variability in total shoulder arthroplasty planning software compared to a control CT-derived 3D printed scapula. Shoulder Elbow 2021;13:268-275. 34659466 10.1177/1758573219888821 PMC8513001

[29] Hall FM: Language of the radiology report: Primer for residents and wayward radiologists. AJR Am J Roentgenol 2000;175:1239-1242. 11044014 10.2214/ajr.175.5.1751239

[30] Eisenberg RL Bankier AA Boiselle PM: Compliance with fleischner society guidelines for management of small lung nodules: A survey of 834 radiologists. Radiology 2010;255:218-224. 20308458 10.1148/radiol.09091556

[31] Srivastava SP Cheng CW Das IJ: The effect of slice thickness on target and organs at risk volumes, dosimetric coverage and radiobiological impact in IMRT planning. Clin Transl Oncol 2016;18:469-479. 26311077 10.1007/s12094-015-1390-z

